# Mechanical Ventilation in COVID-19 Patients: Insights into the Role of Age and Frailty from a Multicentre Observational Study

**DOI:** 10.14336/AD.2022.0127

**Published:** 2022-04-01

**Authors:** Fiona Ecarnot, Paola Rebora, Emanuele Focà, Alberto Zucchelli, Giuseppe Citerio, Maria Grazia Valsecchi, Alessandra Marengoni, Giuseppe Bellelli, for the FRACoViD Team

**Affiliations:** ^1^EA3920, University of Franche-Comté and Department of Cardiology, University Hospital Besançon, Besançon, France.; ^2^Bicocca Center of Bioinformatics, Biostatistics and Bioimaging, School of Medicine and Surgery, University of Milano-Bicocca, Monza, Italy.; ^3^Department of Clinical and Experimental Sciences, University of Brescia, and Division of Infectious and Tropical Diseases, Spedali Civili Hospital, Brescia, Italy.; ^4^Department of Information Engineering, University of Brescia, Brescia, Italy.; ^5^School of Medicine and Surgery, University of Milano - Bicocca, Italy.; ^6^Neuro Intensive Care, San Gerardo hospital, ASST-Monza, Monza Italy.; ^7^Department of Clinical and Experimental Sciences, University of Brescia, Brescia, Italy.; ^8^Acute Geriatric Unit, San Gerardo hospital, Monza, Italy.

**Keywords:** mechanical ventilation, COVID-19, elderly, frailty

## Abstract

In patients with COVID-19, frailty has been shown to better predict outcomes than age alone. We investigated factors associated with mechanical ventilation (MV) during hospitalization for COVID-19 among older adults in a multicentre study during the first two waves in Italy. Using data from the FRACOVID project, we included consecutive patients admitted to the participating centres during the first and second waves. We recorded sociodemographics, comorbidities, time since symptom onset, ventilatory support at admission, and chest X-ray findings. Frailty was assessed using a frailty index (FI). Results are reported as hazard ratios (HR) with 95%CI. 1,344 patients were included; 487 females (36.2%), median age 68 (56; 79) years; 52.4% had hypertension, 10.6% had chronic obstructive pulmonary disease, 15.2% were obese. Median FI was 0.088 (0.03, 0.20), and 67% had bilateral consolidations at admission. Median time since symptom onset was 7 days (4, 10). During hospitalization, 47 patients (3.6%, 95%CI 0.33-13.6%) received MV. Multivariable Cox regression analysis found that the likelihood of intubation decreased with increasing age (HR 0.945 (95%CI 0.921-0.969), p<0.0001), while heart rate >110bpm (HR 3.429 (95%CI 1.583-7.429), p=0.0018), and need for continuous positive airway pressure (CPAP) at admission (HR 2.626 (95%CI 1.330-5.186), p=0.0054) were significantly associated with a greater likelihood of intubation. Older patients are less likely to receive intubation, while those with heart rate >110 bpm and need for CPAP at admission are more likely to receive MV during hospitalization for COVID-19.

In the context of the COVID-19 pandemic, it has been proposed that age alone should not drive clinical decision-making about allocation of healthcare resources [1], with frailty shown to better predict outcomes than age alone, even in people younger than 65 years [2]. Frailty is characterized by increased vulnerability to external stressors, resulting in decreased functional reserve [3]. Prevalence of frailty among patients with COVID-19 is estimated at 45% [4], and during COVID-19 infection, is associated with adverse outcomes [2, 5]. Accordingly, scientific associations and international guidelines [1, 6, 7] (www.nice.org.uk/guidance/ng191/resources/covid19-rapid-guideline-managing-covid19-pdf-51035553326) have emphasized the importance of measuring frailty before deciding whether to admit patients to intensive care (ICU). We investigated factors associated with mechanical ventilation (MV) during hospitalization for COVID-19 among older adults in a multicentre study during the first two waves in Italy.

## METHODS

This analysis used data from the FRACOVID project (NCT04412265). We included consecutive patients admitted to the participating centres during the first (22/02 to 30/06/2020) and second waves (01/07 to 31/12/2020). The study was approved by the Brianza Institutional Review Board (3356-07/08/2020).

The methods of the FRACOVID study have previously been described [8]. Briefly, inclusion criteria were confirmed COVID-19 diagnosis by positive polymerase chain reaction (PCR), or clinical and instrumental evidence; age >18 years; and informed consent. On admission all patients underwent comprehensive geriatric assessment (CGA), including sociodemographics, smoking status, comorbidities, time since symptom onset, ventilatory support at admission, and chest X-ray findings. Frailty was assessed using a frailty index (FI), computed according to the deficit accumulation model, as previously described [2, 8]. The FI yields a continuous score ranging from 0 (no deficits present) to 1 (all deficits present). Patients were followed up until death, discharge or transfer. The ventilatory support required by each patient during hospitalization was established daily together by the attending physician and the intensivist, whereas the decision to intubate the patients for mechanical ventilation was made unilaterally by the intensivist, according to their clinical judgment.

### Statistical analysis

Quantitative data are presented as median [quartile (Q)1, Q3], and categorical variables as number (percentage). Data were compared between patients who received intubation for MV as the maximum ventilatory support during hospitalization, and those receiving all other forms of ventilatory support. Data were compared using the Student t, chi square or Fisher’s exact test, as appropriate. FI was dichotomized according to a threshold which was previous shown to be associated with mortality in this study cohort (0.13) [8]. Variables with a p-value <0.10 by unadjusted analysis were included in Cox regression analysis to identify variables associated with time to intubation. Results are reported as hazard ratios (HR) with 95%CI. Analyses were performed using SAS version 9.4 (SAS Institute Inc., Cary, NC). A p-value <0.05 was considered statistically significant.

## RESULTS

Among 1377 patients included in the FRACOVID cohort, 1344 had available data for maximum ventilatory support and were included. There were 487 females (36.2%), median age was 68 [56; 79] years. More than half had hypertension (52.4%), 10.6% had chronic obstructive pulmonary disease at admission, 15.2% were obese. The median FI was 0.088 [0.03, 0.20]. Two-thirds (67%) had bilateral consolidations on admission chest X-ray. Median time since symptom onset was 7 days [4, 10].

**Table 1:** Results of Cox proportional hazards regression analysis to identify the factors associated with the risk of intubation among older persons hospitalized for COVID-19 during the first two waves in Italy.

Covariable	HR	95% CI	P
Age (per year)	0.945	0.921 - 0.969	<0.0001
Heart rate >110 bpm	3.429	1.583 - 7.429	0.0018
CPAP at admission*	2.626	1.330 - 5.186	0.0054
Fraily Index >0.13	0.806	0.342 - 1.897	0.6210
Female sex	1.037	0.536 - 2.008	0.9134
Second wave (vs first wave)	1.417	0.764 - 2.630	0.2690
Bilateral consolidations on X-ray	2.195	0.742 - 6.492	0.1554

HR, hazard ratio; CI, confidence interval; bpm, beats per minute; CPAP, continuous positive airway pressure. *versus no or non-invasive support.

During hospitalization, 47 patients (3.6%, 95%CI 0.33-13.6%) received MV. By unadjusted analysis, intubated patients were younger, with heart rate >110 bpm, greater oxygen requirements at admission, and more frequent bilateral consolidations on x-ray. Non-mechanically ventilated patients had more comorbidities and higher FI.

Multivariable Cox regression analysis found that the likelihood of intubation decreased with increasing age, while heart rate >110 bpm, and need for continuous positive airway pressure (CPAP) at admission were significantly associated with a greater likelihood of intubation. Frailty, sex, chest X-ray findings and wave were not associated with MV (Table 1).

## DISCUSSION

Our findings show that the likelihood of receiving MV declined with increasing age in this cohort of COVID-19 patients. Elevated heart rate and CPAP at admission were both associated with an increased likelihood of intubation. It has been recommended that age alone should not drive clinical decisions in the COVID setting [1]. Treatment choices should be examined in light of chances of recovery and expected quality of life, in agreement with international guidelines [1, 7], which recommend that frailty should also be assessed, as a more informative and less discriminatory measure than age [9]. In our study, frailty was not related to MV, suggesting that treatment decisions may have been guided only by age and severity of disease. Perhaps intensivists sought to ration care by reserving MV for the youngest, and therefore, likely most robust patients.

Our findings indicate that there is room to improve the pervasive temptation to use age as the only criterion for allocation of healthcare resources. It is noteworthy that the median FI of the study cohort was very low, indirectly suggesting that patients with mild or higher frailty levels were not even assessed with a view to receiving MV. This hypothesis is supported by the observation that only 3.6% received MV in this cohort. There is thus potential for selection bias. In a context of limited resources, urgent decision-making, and demand-supply mismatch, as was the case in the Lombardy Region during the first wave, this approach may seem understandable, perhaps unavoidable, even if not acceptable overall. Nevertheless, after the first wave, there was a surge in the number of ICU beds in our Region [10], expanding capacity to cater for larger numbers of candidates for MV. Furthermore, during the second wave, other management strategies (such as transfer of people requiring intubation to other ICUs) might have been employed. To support the plausibility of different health-policy strategies in caring for older patients with COVID-19, a recent cross-sectional study among public and private hospitals in France found that, among 9885 patients admitted to ICU during the COVID-19 pandemic, 47% were aged 65-79 years and 4.8% were aged 80 years and above [11]. There may be potential selection bias, whereby older patients had a loss-of-opportunity for care, with a limitation on the intensity of therapy [12]. Intensivists should be aware of the potential for ageism in selecting treatment options based solely on chronological age and should be encouraged to measure frailty systematically in their patients, to improve prediction of short-term outcomes.

We also observed that patients with a heart rate >110 bpm were more likely to be intubated, congruent with previous reports that abnormal heart rate is observed in 6 to 7% of hospitalized COVID-19 patients [13]. Heart rate variability is a key marker of inflammatory activity and immune system activation [14]. Similarly, patients who required CPAP at admission were more likely to progress to intubation. This is line with previous reports that the severity of respiratory failure at admission is associated with disease progression and COVID-19-associated mortality [15]. Similarly, in a study of 868 patients receiving MV in the ICU for COVID-19, a failed attempt at non-invasive positive pressure ventilation prior to orotracheal intubation was associated with an almost 2-fold increase in 6-month mortality (odds ratio 1.878 (95% CI 1.124-3.140)) [16].

## Conclusion

Our findings indicate that older patients are less likely to receive intubation, while those with heart rate >110 bpm and need for CPAP at admission are more likely to receive MV as maximum level of ventilatory support during hospitalization for COVID-19.
